# β‐asarone inhibits the migration, invasion, and EMT of bladder cancer through activating ER stress

**DOI:** 10.1002/cam4.6059

**Published:** 2023-06-12

**Authors:** Bo Liu, Weichao Dan, Yi Wei, Yan Zhang, Chi Wang, Yuzeshi Lei, Tao Hou, Yong Zhang, Ye Gao

**Affiliations:** ^1^ Department of Surgical Oncology The First Affiliated Hospital of Xi'an Jiaotong University Xi'an P. R. China; ^2^ Department of Urology The First Affiliated Hospital of Xi'an Jiaotong University Xi'an P. R. China; ^3^ Key Laboratory of Environment and Genes Related to Diseases, Ministry of Education Xi'an P. R. China; ^4^ Key Laboratory for Tumor Precision Medicine of Shaanxi Province The First Affiliated Hospital of Xi'an Jiaotong University Xi'an P. R. China; ^5^ Department of Emergency The First Affiliated Hospital of Xi'an Jiaotong University Xi'an P. R. China

**Keywords:** ATF6, EMT, ER stress, invasion, migration, β‐asarone

## Abstract

**Background:**

β‐asarone (β‐as), a compound extracted from Acorus calamus, has been found to have anticancer effects on a variety of human cancers. However, the potential effect of β‐as on bladder cancer (BCa) remains unknown.

**Methods:**

After exposure to β‐as, migration, invasion, and epithelial‐mesenchymal transition (EMT) of BCa were determined by wound healing, transwell, and Western blot assays. Expression of proteins involved in the EMT and ER stress were explored by Western blot assays. Nude mouse xenograft model was served as the model system in vivo.

**Results:**

The migration, invasion, and EMT of BCa were significantly inhibited after β‐as treatment. Further experiments revealed that endoplasmic reticulum (ER) stress is involved in β‐as‐mediated metastasis inhibition. In addition, β‐as significantly up‐regulated activating transcription factor 6 (ATF6), a branch of ER stress, and promoted its Golgi cleavage and nuclear localization. ATF6 silencing attenuated β‐as‐mediated metastasis and EMT inhibition in BCa cells.

**Conclusion:**

Our data suggests that β‐as inhibits migration, invasion, and EMT of BCa by activating the ATF6 branch of ER stress. Thus, β‐as represents a potential candidate for BCa treatment.

## Graphical abstract

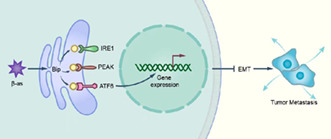

## 1 INTRODUCTION

Bladder cancer (BCa) is one of the most common malignancies of the urinary system. Approximately 900,000 new cases and 250,000 BCa cancer‐related deaths are reported worldwide every year.^1^ In China, BCa‐related mortality ranked among the top ten cancer‐related deaths in 2016 and accounted for over a tenth of all BCa‐related deaths worldwide.^2^ BCa significantly affects the patient's quality of life. Non‐muscle‐invasive BCa, which accounts for approximately 75% of all new BCa cases, has a high rate of recurrence, whereas muscle‐invasive BCa, which accounts for the remaining cases, has a high degree of malignancy.^3^ Owing to its high degree of malignancy and recurrence rate, obtaining satisfactory prognosis with surgical treatment alone or combined chemotherapy remains a challenge.^4^ Furthermore, a significant number of patients experience adverse effects following chemotherapy.^4^ Although the emerging bacillus Calmette‐Guerin (BCG) infusion therapy has improved the prognosis of patients with BCa in the recent years, there is an urgent need for new treatment regimens for the subset of patients who are insensitive to BCG.^5^ Over the past decade, the anticancer effects of several Chinese herbal medicines have been reported, and these are expected to contribute to the development of new treatments for BCa.

β‐asarone (β‐as), a compound extracted from the traditional Chinese medicinal herb *Acorus calamus*, is rapidly absorbed and distributed in the body.^6^ It quickly crosses the blood–brain barrier (BBB) and is used to treat Alzheimer's disease.^7^ In addition to its application in the treatment of neurological diseases, increasing number of studies have confirmed that β‐as has antitumor effects, such as in lung cancer,^8^ gastric cancer,^9^ colorectal cancer,^10^ glioblastoma,^11^ and others. Metastasis plays a crucial role in the malignant progression of cancers. Therefore, many anticancer studies have focused on inhibiting the invasion and migration of tumor cells.^12^, ^13^ Several studies have shown that β‐as suppresses the invasion and migration of lung cancer and glioblastoma cells.^8^, ^11^ Nevertheless, no study has yet evaluated the antitumor effect of β‐as in BCa, or examined whether it mitigates invasion and migration of BCa cells.

Adverse conditions such as starvation, hypoxia, increased levels of free radicals, and excessive acid result in the accumulation of misfolded and unfolded proteins in the endoplasmic reticulum (ER) resulting in ER stress, which activates the process of unfolded protein response (UPR).^14^ Davis et al. demonstrated that ER stress stimulates immunological response, regulates metabolism, and promotes autophagy and apoptosis.^15^ A previous study showed that activating transcription factor 6 (ATF6), inositol‐requiring enzyme type 1 (IRE1), and protein kinase R (PKR)‐like ER kinase (PERK) signaling pathways are involved in the activation of ER stress.^16^ ATF6, located in the ER, is activated by intramembrane proteolysis in response to ER stress, and is a major regulator of organogenesis and tissue homeostasis.^17^ Although the ATF6, IRE1, and PERK signaling pathways are parallel branches that induce ER stress, activated ATF6 can positively regulate other branches, thereby intensifying ER stress further.^18^, ^19^ Studies have shown that ATF6 promotes the metastasis of cervical^20^ and breast cancers.^21^ The transformation of epithelial cells into mesenchymal cells, which plays a vital role in tumor invasion and migration, is known as epithelial‐mesenchymal transformation (EMT).^22^ ER stress regulates cancer cell proliferation and metastasis,^23^ and activation of ER stress has been shown to suppress the metastasis of breast^24^ and gastric cancers.^25^ However, to the best of our knowledge, no study has examined the relationship between ER stress and invasion or migration in BCa.

This study aimed to investigate the effect of β‐as on metastasis and EMT of BCa, and to identify the underlying molecular mechanism. The results suggest that β‐as inhibits migration, invasion, and EMT of BCa by activating the ATF6 branch of ER stress.

## 2 MATERIALS AND METHODS

### 2.1 Reagents and antibodies

β‐asarone (cat. no. HY‐N1501) was purchased from MedChemExpress and dissolved in dimethyl sulfoxide (DMSO). Tauroursodeoxycholic acid (TUDCA; cat. no. HY‐19696) was purchased from MedChemExpress. Primary rabbit antibodies against ATF6 (cat. no. ab37149; 1:2000), GRP78/BIP (cat. no. ab21685; 1:2000), and IRE1 (cat. no. ab124945; 1:2000), and secondary goat anti‐rabbit IgG (cat. no. ab205718; 1:2000) were purchased from Abcam. Primary rabbit antibodies against E‐cadherin (cat. no. 14472S; 1:2000), N‐cadherin (cat. no. 13116T; 1:2000), vimentin (cat. no. 46173SF; 1:2000), PERK (cat. no. 72908S; 1:2000), GAPDH (cat. no. 5174T; 1:5000), histone (cat. no. 4499T; 1:2000), and β‐actin (cat. no. 4970T; 1:5000) were purchased from Cell Signaling Technology Inc.

### 2.2 Cell culture

Human BCa cell lines 253J and 5637 were obtained from American Type Culture Collection and cultured in Roswell Park Memorial Institute‐1640 (RPMI‐1640) medium (Gibco; Thermo Fisher Scientific Inc.) and Dulbecco's modified Eagle's medium (DMEM, high glucose medium, Gibco; Thermo Fisher Scientific Inc.) supplemented with 10% fetal bovine serum (Gibco; Thermo Fisher Scientific Inc.). Both the culture media were also supplemented with 1% (volume) of 0.1 mg/mL streptomycin and 100 U/mL penicillin (Gibco; Thermo Fisher Scientific Inc.).

### 2.3 MTT assay

253J and 5637 cells were plated in 96‐well plates at a density of 4.0 × 10^4^ cells/mL. Subsequently, the cells were treated with different concentrations of β‐as for 24 h or 48 h, and then, 200 mL MTT (5 mg/mL) solution was added into each well and incubated for 4 h. The formazan crystals were dissolved in DMSO by oscillating the 96‐well plates was on a mini shaker for 10 min. The optical density was read at 490 nm using a microplate reader (Bio‐Rad Laboratories Inc.).

### 2.4 Transwell migration and invasion assays

Following digestion with Trypsin‐EDTA (0.25%) (Gibco; Thermo Fisher Scientific Inc.), the cells were resuspended in serum‐free medium at a concentration of 5 × 10^5^/mL. For invasion assay, a 50 μL mixture of DMEM (high glucose)/Matrigel™ (9/1) (Sigma‐Aldrich; Merck KGaA) was plated in the upper chamber. RPMI‐1640 supplemented with 10% FBS was added to the lower chamber, and then, BCa cells were plated in the upper chamber. Following incubation at 37°C for 24 h, the transwell chamber was washed with phosphate buffered saline (PBS) thrice and then fixed with 4% paraformaldehyde at room temperature for 15 min. The chambers were washed again with PBS thrice and stained with 0.1% crystal violet for 15 min. Five fields of view (magnification, ×200) were selected randomly and studied under an inverted microscope. All the steps for the migration assay, except for the addition of DMEM/Matrigel™, were the same as described in the invasion assay protocol.

### 2.5 Wound healing assay

BCa cells were allowed to grow to 75%–85% confluence in 6‐well plates. The cell monolayer was then gently scratched with a 200 μL‐pipette tip. The cells were washed with PBS (Gibco; Thermo Fisher Scientific Inc.) twice and then cultured in serum‐free DMEM (high glucose) containing β‐as or not. The cells in the wells were observed under an inverted microscope at 0, 12, and 24 h after scratching, and images were captured. The gap distance filled by the cells was measured using ImageJ 1.48v software (National Institute of Health).

### 2.6 Western blot analysis

Following treatment with β‐as for 24 h, BCa cell lines were lysed with radioimmunoprecipitation assay buffer (50 mM Tris, 150 mM NaCl, 0.1% SDS, 1% NP40 and 0.5% sodium deoxycholate; pH 7.4) containing proteinase inhibitors (cat. no. 04693132001; Sigma‐Aldrich; Merck KGaA). The denatured protein was separated by 12% sodium dodecyl sulfate polyacrylamide gel electrophoresis (SDS‐PAGE) and then transferred to polyvinylidene difluoride (PVDF) membranes. The non‐specific sites on the membrane were blocked with 5% skim milk for 1 h, and the membrane was then incubated with primary antibodies against ATF6 (1:2000), GRP78/BIP (1:2000), and IRE1 (1:2000), E‐cadherin (1:2000), N‐cadherin (1:2000), Vimentin (1:2000), PERK (1:2000), GAPDH (1:5000), histone (1:2000), and β‐actin (1:5000) overnight at 4°C. Following three washes with TBST (15 min each), the membranes were incubated with the secondary rabbit anti‐Goat IgG antibody (1:2000) for 1 h. Subsequently the membranes were washed three times with TBST. Ultimately, the signals were detected using the Clarity™ Max Western ECL substrate, followed by exposure to X‐ray films.

### 2.7 Total RNA isolation and quantitative RT‐PCR

Total RNA was isolated from cells in the β‐treated and control groups using the Tsingke RNA Extraction Kit according to the manufacturer's instructions. The concentration of total RNA was determined using a microplate assay (Bio‐Rad Laboratories Inc.). The cDNA was reverse transcribed using 20 μL of total RNA and components from the High‐capacity cDNA Reverse Transcription Kit (cat. no. 4368814; Thermo Fisher Scientific Inc.) according to the manufacturer's instructions. The relative expression levels of ATF6, IRE1, PERK, and BIP mRNAs were analyzed by realtime qPCR using 20 μL of cDNA and reagents from the 2× T5 fast qPCR Mix (SYBR Green I [cat. no. TSE202; Tsingke Biotechnology]).

### 2.8 Immunofluorescence

BCa cells were seeded in a 12‐well plate with coverslips, and treated with β‐as or not for 24 h. The coverslips were transferred to glass slides and fixed at room temperature for 15 min with 4% paraformaldehyde. The slides were washed with PBS thrice, and the cells were permeabilized with 0.5% Triton X‐100 solution followed by incubation with primary antibody against ATF6 (1:2000) at 4°C overnight. Following three washes with PBS, the cells were incubated with FITC‐labeled goat anti‐rabbit IgG (H + L) (cat. no. A0562; 1:200; Beyotime) at room temperature for 1 h and counterstained with 4′, 6′‐diamidino‐2‐phenylindole DAPI (1 μg/mL) for 5 min in the dark.

### 2.9 Preparation of cytoplasmic and nuclear extracts

BCa cells were plated in plates and treated with β‐as or not for 24 h. Subsequently, cytoplasmic and nuclear proteins were extracted using the Nuclei EZ Prep Nuclei Isolation kit according to manufacturer's protocol. Expression of ATF6, GAPDH, and histone was verified by Western blot analysis.

### 2.10 Small interfering (si) RNA transfection

SiRNA targeting ATF6 (si‐ATF6) was purchased from Shanghai GenePharma Co. Ltd. The si‐ATF6 sequence was 5′‐ GGAGACAGCAACGUAUGAUTT‐3′, whereas the sequence of the corresponding negative control siRNA (si‐NC) was 5′‐UUCUCCGAACGUGUCACGUTT‐3′. The transfection reagent was made by mixing siRNA and Lipofectamine® 2000 (Invitrogen; Thermo Fisher Scientific Inc.) in Opti‐MEM® (Gibco; Thermo Fisher Scientific Inc.). After incubating the mixture for 15 min, the transfection reagents were used to transfect cells. RT‐qPCR and Western blot analyses were used to detect the transfection efficiency after 48 h.

### 2.11 Xenograft animal model

Balb/c male nude mice (4‐weeks‐old; *n* = 10, 15–20 g) were procured from the Laboratory Animal Center of the Xi'an Jiaotong University. All animal care and experiments were authorized by the Institutional Animal Care and Use Committee of the Xi'an Jiaotong University. The health and behavior of the nude mice were monitored daily. The mice were maintained in specific pathogen‐free rooms under 12‐h light/dark cycle at 22–25°C with free access to water and food. BCa 5637 cells were suspended in serum‐free culture medium containing matrigel to the density of 5.0 × 10^6^/mL, and 100 μL cell suspension was injected subcutaneously on left and right sides of the mice. Once the tumor volume reached 100–150 mm^3^, the mice were divided into control (*n* = 5) and β‐as treatment groups (*n* = 5, 50 mg/kg, intraperitoneal injection, every 2 days for 2 weeks). The mice were treated, and the tumor volume was calculated as follows: volume (mm^3^) = 0.5 × (length) × (width)^2^ once every 2 days. Tumor was excised and collected after 16 days, and the mice were sacrificed with carbon dioxide (CO_2_) with a CO_2_ emission of 17.5%/min. The mice were exposed to CO_2_ and monitored until the breathing stopped completely for 10 min. The excised tumor was embedded in paraffin, and the relevant indexes were examined by immunohistochemistry and Western blot assays. A second batch of ten (4‐weeks‐old) Balb/c male nude mice were divided into two groups to generate the metastatic model. Each mouse was injected with 5637 cell suspension by tail vein injection and treated as described above. After 30 days, the metastases in the lung were examined and analyzed by H&E staining.

### 2.12 Statistical analysis

All statistical analyses were performed using GraphPad Prism 5.2 software (GraphPad Software Inc.). All data are presented as the mean ± standard deviation (SD) of three independent experiments. A Student's *t*‐test was used for the comparisons between two groups, and the statistical significance was set at *p* < 0.05.

## 3 RESULTS

### 3.1 β‐as suppresses migration, invasion, and EMT of BCa cells

**FIGURE 1:**
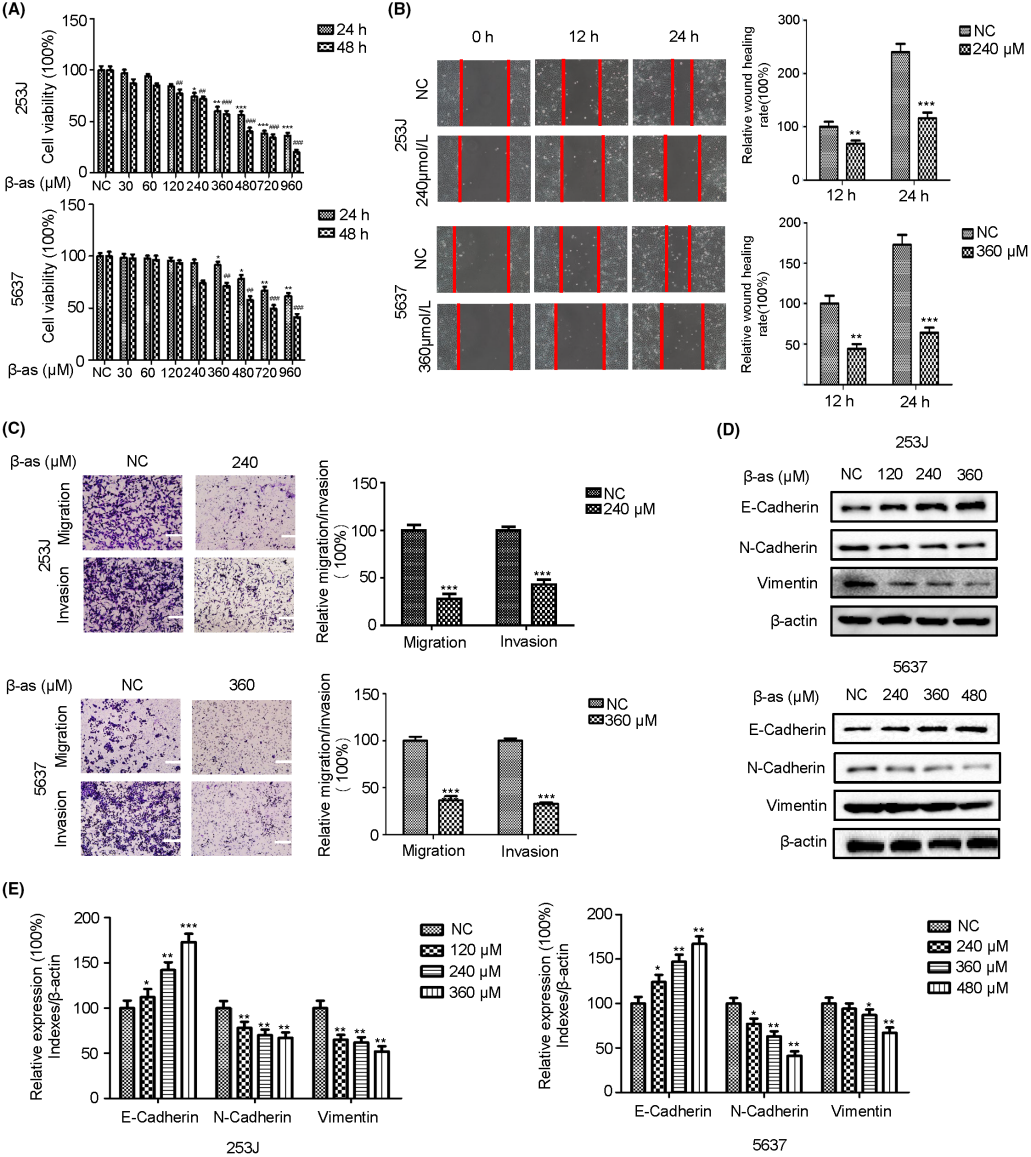
β‐asarone (β‐as) suppresses migration, invasion, and epithelial‐mesenchymal transition (EMT) of bladder cancer (BCa) cells. (A) Viability of 253J and 5637 cells was assessed using MTT assay. Cells were treated with different concentrations of β‐as (0, 30, 60, 120, 240, 360, 480, 720, and 960 μM) for 24 and 48 h. *p < 0.05, **p < 0.01, ***p < 0.001, and ^##^ *p* < 0.01, ^###^ *p* < 0.001. (B) Wound healing assay was performed to determine the relative wound healing rates of 253J and 5637 cells treated with β‐as (240 or 360 μM) for 24 h. ***p < 0.001. (C) Transwell migration and invasion assays were performed to determine the relative migration and invasion rates of 253J and 5637 cells treated without or with β‐as (240 or 360 μM). Magnification, ×100. Scale bar, 200 μm. ***p < 0 0.001. (D, E) 253J and 5637 cells were treated with different concentrations (0, 120, 240, and 360 μM and 0, 240, 360, and 480 μM, respectively) of β‐as for 24 h. Western blotting was performed to detect the protein expression of E‐cadherin, N‐cadherin, and vimentin. *p < 0.05, **p < 0.01, ***p < 0.001. All data are presented as the mean ± standard deviation (SD) of three independent assays.

To examine the inhibitory effect of β‐as on BCa cells, we performed MTT assay following treatment of 253J and 5637 cells with different concentrations of β‐as for 24 or 48 h. The results indicated that the inhibitory effect of β‐as on proliferation of BCa cells was concentration‐ and time‐dependent, with IC_50_ values of 684 and 982 μM in 253J and 5637 cells, respectively, at 24 h (Figure 1). Wound healing and transwell assays were used to examine the effect of β‐as on the migration and invasion ability of BCa cells. The results illustrated that β‐as significantly inhibited the migration and invasion ability of BCa cells (Figure 1B,C). To determine whether β‐as affects EMT process in BCa cells, we treated the cells with various concentrations of β‐as and determined the expression of EMT‐related markers (E‐cadherin, N‐cadherin, and vimentin) using Western blot analysis. As shown in Figure 1D,E, β‐as up‐regulated the expression of E‐cadherin and down‐regulated the expression of N‐cadherin and vimentin in BCa cells in a concentration‐dependent manner, suggesting that β‐as inhibits the EMT process in BCa cells. The above results suggest that β‐as suppresses the migration, invasion, and EMT of BCa cells.

### 3.2 β‐as activates ER stress in BCa cells

**FIGURE 2:**
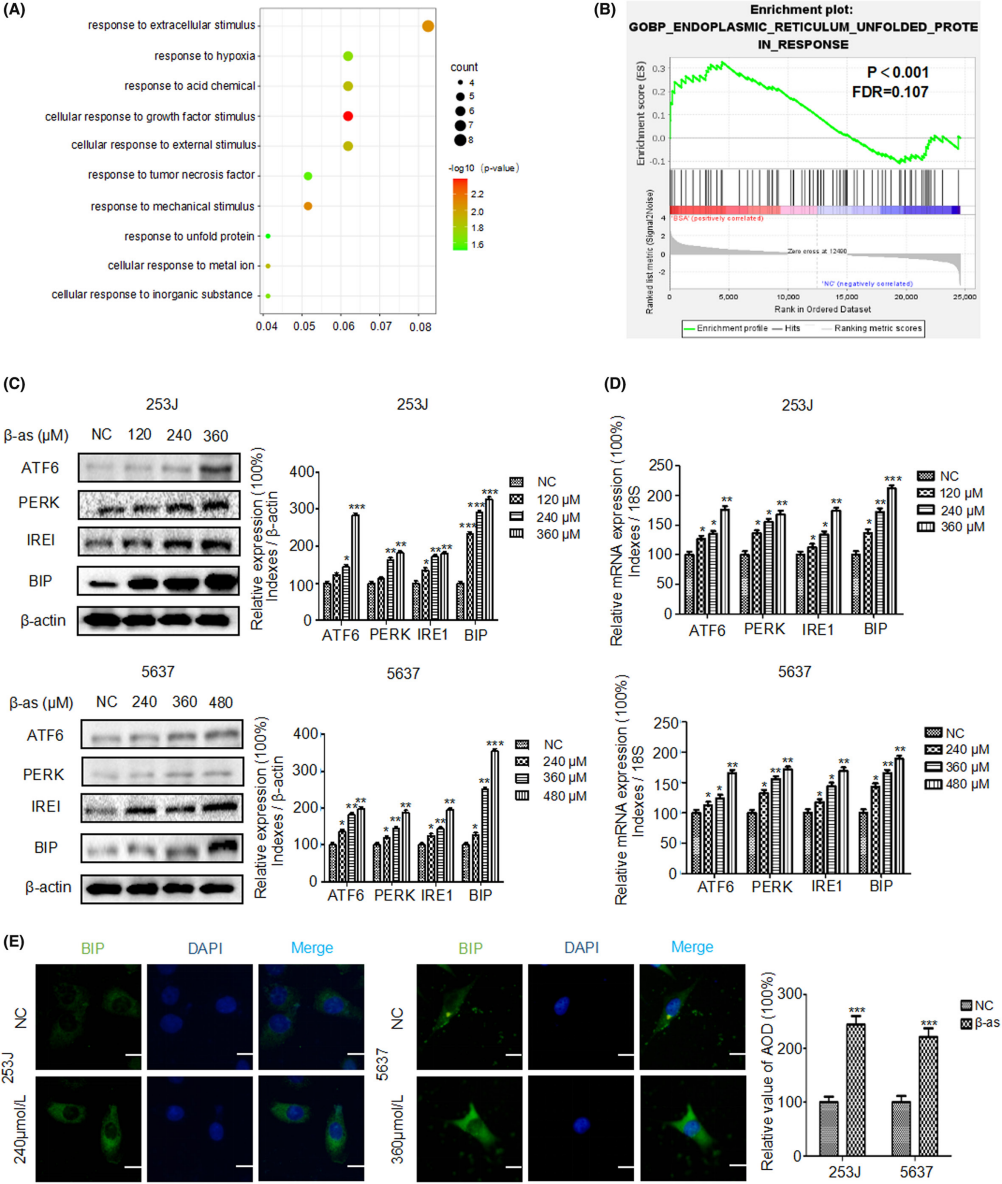
β‐asarone (β‐as) s activates endoplasmic reticulum (ER) stress in BCa cells. (A) RNA‐seq data for 5637 cells treated with β‐as. BCa 5637 cells were treated with β‐as (240 μM), and total RNA was extracted for sequence analysis. (B) Analysis of gene ontology biological process (GOBP) database revealed enrichment of ER unfolded protein response. *p* < 0.001. (C) Expression of ER stress‐related protein markers (ATF6, PERK, IRE1, and BIP) in 253J and 5637 cells treated with different concentrations of β‐as (0, 120, 240, and 360 μM and 0, 240, 360, and 480 μM, respectively) for 24 h was analyzed by Western blotting. *p < 0.05, **p < 0.01, ***p < 0.001. (D) mRNA levels of ER stress‐related markers in 253J and 5637 cells treated with different concentrations (0, 120, 240, and 360 μM and 0, 240, 360, and 480 μM, respectively) of β‐as for 24 h were measured by q‐PCR. *p < 0.05, **p < 0.01, ***p < 0.001. (E) Expression of BIP in 253J and 5637 cells that were mock‐treated or treated with β‐as (240 or 360 μM) was analyzed using immunofluorescence. Scale bar, 50 μm. ***p < 0.001. All data are presented as the mean ± SD of three independent assays.

To identify the signaling pathways activated following β‐as treatment, we analyzed RNA‐seq of β‐as‐treated 5637 cells and mapped the gene ontology biological process (GOBP) database. We found the response to ER UPR was enriched (Figure 2A,B). To investigate whether β‐as plays a role in ER stress in BCa cells, 253J/5637 cells were treated with different concentrations of β‐as, followed by examination of the expression levels of ER stress‐related markers (ATF6, PERK, IRE1, and BIP) by Western blotting. As shown in Figure 2C, β‐as up‐regulated the expression of ATF6, IRE1, and BIP in a concentration‐dependent manner. Similarly, RNA was extracted from 253J/5637 cells and reverse transcribed. The ER stress‐related markers ATF6, IRE1, PERK, and BIP were amplified and quantified in real time. The results showed that β‐as activates ER stress‐related markers at the transcriptional level (Figure 2D). Immunofluorescence assay demonstrated that the expression level of BIP, an indicator of ER stress, increased following β‐as treatment (Figure 2E). These results suggest that β‐as activates ER stress in BCa cells.

### 3.3 β‐as suppresses migration, invasion, and EMT of BCa cells by activating ER stress

**FIGURE 3:**
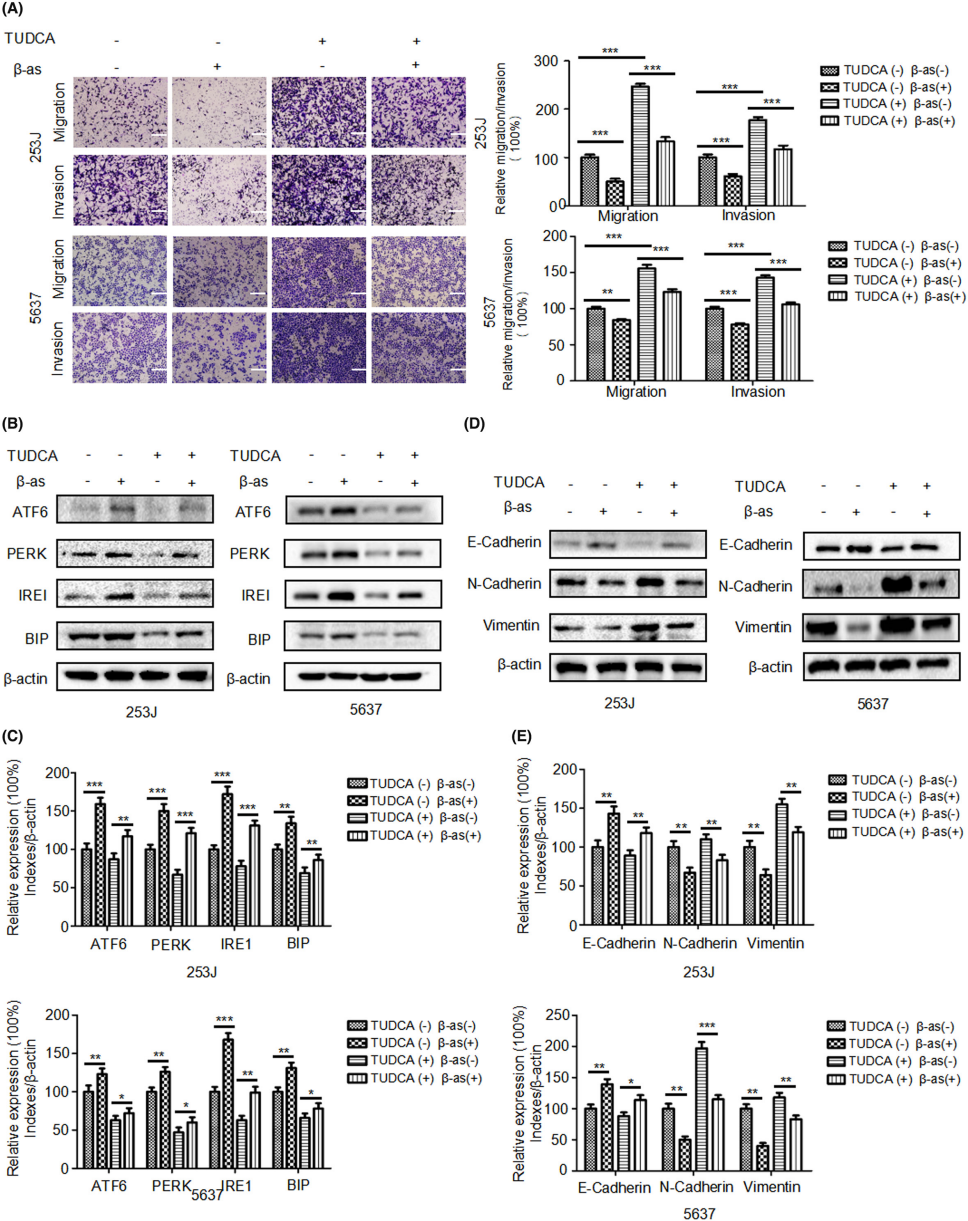
β‐asarone (β‐as) suppresses migration, invasion, and epithelial‐mesenchymal transition (EMT) of BCa cells via activating ER stress. (A) 253J and 5637 were treated without or with β‐as (240 or 360 μM) in the presence or absence of tauroursodeoxycholic acid (TUDCA; 10 μM) for 24 h and subjected to transwell migration and invasion assays, Magnification, ×100. Scale bar, 200 μm. ***p < 0.001. (B, C) Western blot analysis of the expression of ER stress‐related protein markers (ATF6, PERK, IRE1, and BIP) in 253J and 5637 cells treated as described in (A). *p < 0.05, **p < 0.01, ***p < 0.001. (D, E) Western blot analysis of the protein expression of E‐cadherin, N‐cadherin, and vimentin in 253J and 5637 cells treated as described in (A). *p < 0.05, **p < 0.01, ***p < 0.001. All data are presented as the mean ± SD of three independent assays.

To verify that β‐as inhibits migration, invasion, and EMT of BCa cells by activating ER stress, we used TUCDA, the ER stress inhibitor, to inhibit ER stress in BCa cells during β‐as treatment. The results of both transwell migration and invasion assays showed that, compared with the control group, the number of cells passing through the upper chamber was significantly higher in the group treated with TUDCA alone. In addition, compared with the group treated with both TUDCA and β‐as, the number of cells passing through the upper chamber was reduced in the group treated with β‐as only (Figure 3A), which suggests that β‐as activates ER stress to suppress the migration and invasion of BCa cells. Western blotting results (Figure 3B–E) also demonstrated that β‐as reduced the inhibitory effect of TUCDA on ER stress, and further inhibited EMT of BCa cells. This suggests that β‐as suppresses the EMT of BCa cells by activating ER stress. Thus, these results confirm that β‐as inhibits the migration, invasion, and EMT of BCa cells by activating ER stress.

### 3.4 β‐as suppresses migration, invasion, and EMT of BCa cells through the ATF6 branch of ER stress

**FIGURE 4:**
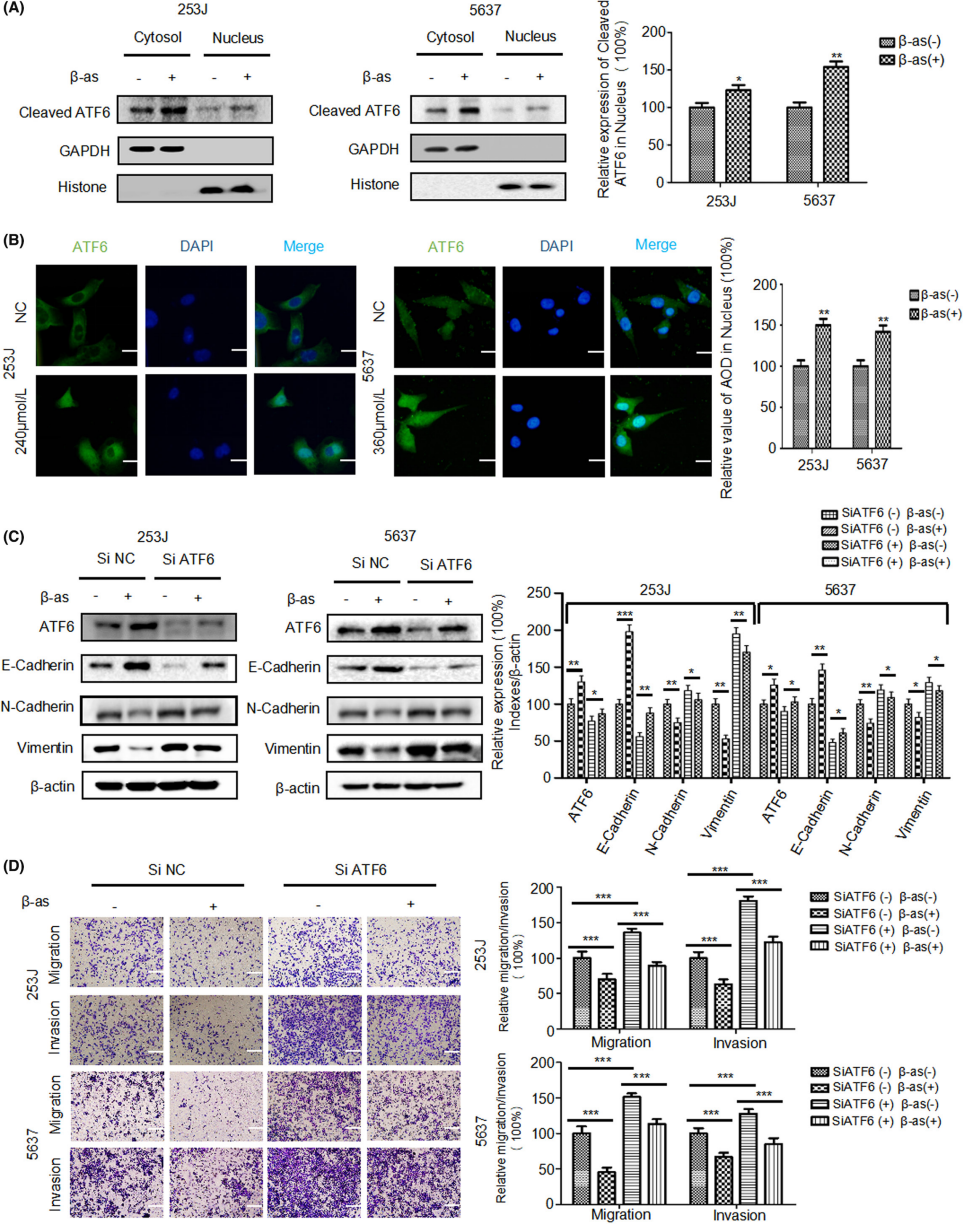
β‐as suppresses migration, invasion, and EMT of BCa cells through the ATF6 branch of ER stress. (A) 253J and 5637 cells were treated with β‐as (240 or 360 μM) for 24 h, and cytosolic and nuclear protein level of ATF6 were analyzed by Western blotting. *p < 0.05, **p < 0.01. (B) Immunofluorescence analysis of the expression of ATF6 in 253J and 5637 cells that were mock‐treated or treated with β‐as (240 or 360 μM), Scale bar, 50 μm. **p < 0.01. (C) 253J and 5637 cells without or with ATF6 knockdown were treated without or with β‐as (240 or 360 μM) for 24 h. Protein expression of ATF6, E‐cadherin, N‐cadherin, and vimentin was analyzed in the cells by Western blotting. *p < 0.05, **p < 0.01, ***p < 0.001. (D) Relative migration and invasion rates of 253J and 5637 cells treated as described in (A) was determined using transwell migration and invasion assays. Magnification, ×100. Scale bar, 200 μm. ***p < 0.001. All data are presented as the mean ± SD of three independent assays.

The transcription factor, ATF6, functions mainly after entering the nucleus. As shown in Figure 4A, analysis of ATF6 expression in the cytoplasmic and nuclear extracts confirmed that β‐as plays a role in promoting ATF6 entry into the nucleus in BCa cells. Similarly, immunofluorescence assay was performed to examine the effect of β‐as in the post‐entry role of ATF6. The results (Figure 4B) confirmed that β‐as markedly promotes the post‐entry role of ATF6 in BCa cells. To confirm that β‐as suppresses the migration, invasion, and EMT of BCa cells through the ATF6 branch of ER stress, we used siATF6 to knockdown the expression of ATF6 in 253J and 5637 cells. 253J and 5637 cells successfully transfected with siNC and siATF6 and treated with TUDCA or β‐as. Western blotting results showed that knockdown of ATF6 expression reduced the β‐as‐mediated inhibition of EMT in BCa cells (Figure 4C). This was consistent with the transwell migration and invasion assay results which showed that, in the absence of β‐as treatment, compared with siNC group, the number of cells crossing the upper chamber was markedly higher in the siATF6 group. In the β‐as treated cells, compared with siNC group, the number of cells passing through the upper chamber was markedly increased in the siATF6 group (Figure 4D), which confirmed that β‐as activates the ATF6 branch of ER stress to suppress the metastasis of BCa cells. These results confirmed that β‐as suppresses migration, invasion, and EMT of BCa cells by activating the ATF6 branch of ER stress.

### 3.5 Antitumor effects of β‐as on BCa cells in vivo

**FIGURE 5:**
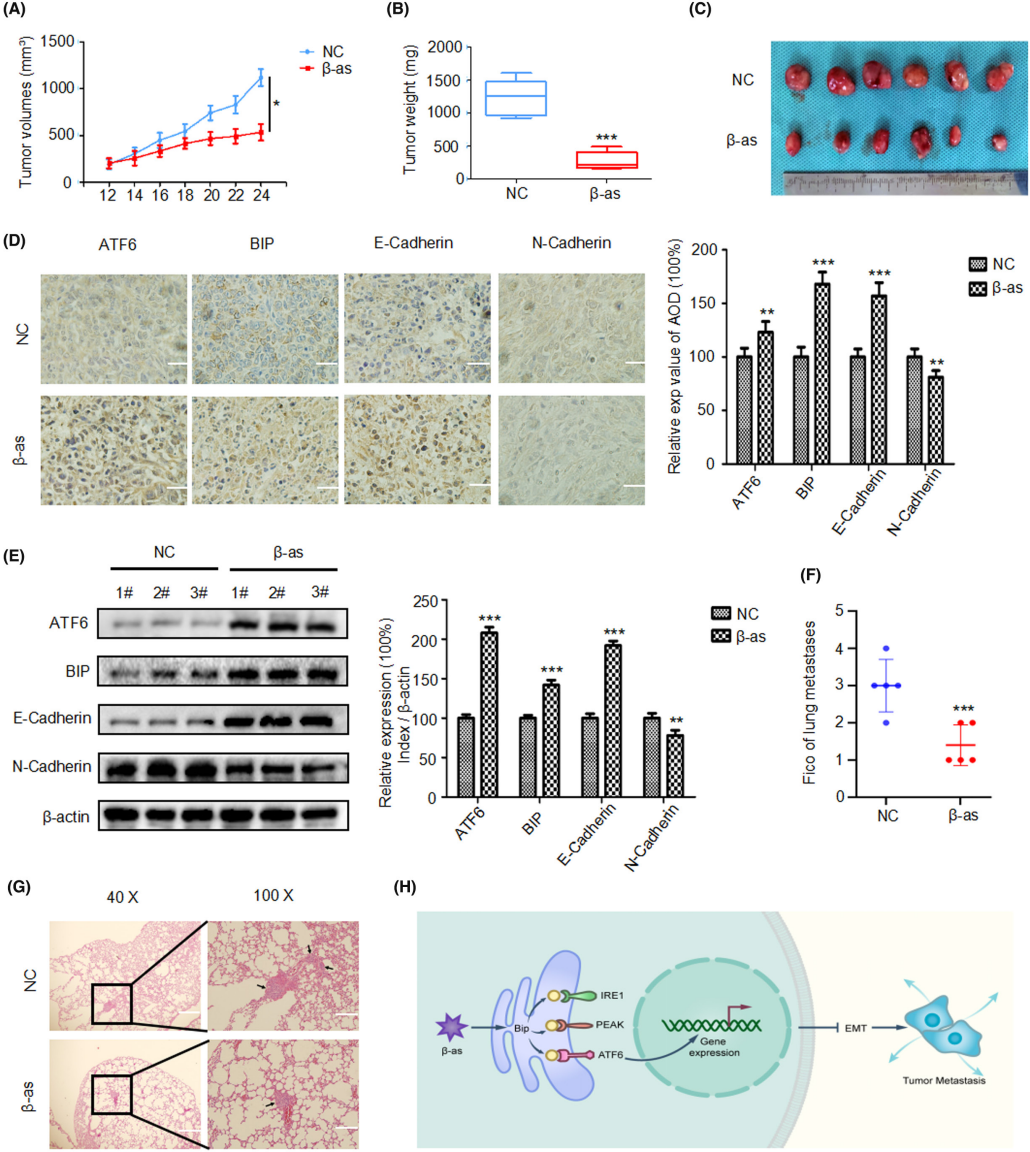
Antitumor effects of β‐asarone (β‐as) on BCa cells in vivo. (A) Tumor volume was measured every 2 days in the control and β‐as groups. (B, C) Subcutaneous xenograft tumors were dissected following treatment without or with β‐as for 16 days and weighed. ***p < 0.001 (D) Immunohistochemical analysis of the expression of ATF6, BIP, E‐cadherin, and N‐cadherin in the tumor tissues. Scale bar, 20 μm. **p < 0.01, ***p < 0.001. (E) Western blot analysis of the expression of ATF6, BIP, E‐cadherin, and N‐cadherin in tumor tissues. **p < 0.01, ***p < 0.001. (F, G) Pathological examination of sections of metastatic lung tissues from the control and β‐as‐treated groups following hematoxylin–eosin staining, Scale bar, left 25 μm, right 10 μm. ***p < 0.001. (H) Schematic diagram illustrating the effects of β‐as in BCa based on the data obtained from this study. β‐as inhibits the migration, invasion, and EMT of BCa cells by activating ER stress. All data are presented as the mean ± SD of three independent assays.

To examine the antitumor effects of β‐as on BCa cells in vivo, xenograft and metastatic tumor models were constructed by injecting 5637 cells into mice subcutaneously and intravenously, respectively. The control group was administered intraperitoneal injection of normal saline, whereas the treatment group was administered intraperitoneal injection of β‐as. As illustrated in Figure 5A–C, the tumors in the β‐as treated group grew more slowly, and were smaller in size and lighter than those in the control group. Immunohistochemistry and Western blotting results confirmed that β‐as up‐regulated the expression of ATF6, BIP, and E‐cadherin, and down‐regulated the expression of N‐cadherin in the tumor tissues of mice (Figure 5D,E). In addition, metastatic tumors in the lungs were markedly lower in the β‐as‐treated group than in the control group (Figure 5F,G). Thus, these data suggest that β‐as inhibits the migration, invasion, and EMT of BCa by activating ER stress (Figure 5H). Furthermore, the results demonstrated the antitumor effects of β‐as on BCa cells in vivo.

## 4 DISCUSSION

With the rapid advancements in science and technology in recent years, scientists have been exploring new cancer treatment regimens and anticancer drugs in an effort to improve the outcome of cancer therapies. Several researchers have focused their attention on traditional Chinese medicine, and some herbal extracts with anticancer activity have been instituted in commonly recognized treatment. β‐as, distilled from *A. calamus*, is one such newly discovered extract that shows excellent anti‐inflammatory and anticancer properties.^26^ β‐as has been shown to possess anticancer activity in lung cancer,^9^ colorectal cancer,^10^ and glioblastoma.^11^ Our results demonstrate that β‐as inhibits migration of BCa cells in vitro and metastasis in vivo. Using a mouse model, Pastushenko et al. showed that EMT plays a crucial role in the occurrence and metastasis of cancer.^27^ Furthermore, Li et al. found that β‐as inhibits EMT in human glioma cells by suppressing the splicing factor HnRNP A2/B1.^28^ Our Western blotting results indicated that β‐as up‐regulates the expression of E‐cadherin and down‐regulates the expression of N‐cadherin and vimentin in BCa, which suggests that β‐as suppresses the EMT of BCa cells. However, the mechanisms involved need to be investigated further.

ER stress is a protective mechanism used by cells to deal with adverse external conditions, such as inhibiting tumor progression.^29^ Existing evidence have confirmed that ER stress exerts anticancer effects through various mechanisms. Knockdown of ERLIN2 (ER stress‐related gene) expression resulted in enhanced invasion and migration ability of breast cancer cells.^30^ In addition, CHOP, a downstream target protein of ER stress, has been confirmed to induce apoptosis of cancer cells.^31^ Previous in vitro and in vivo studies have confirmed that activation of ER stress inhibits progression of EMT, which promotes tumor metastasis.^32^ Bandyopadhyaye et al. demonstrated that ER stress activation and calcium ion release increased the level of NDRG1 transcription to inhibit the colonization of metastatic prostate cancer cells in lungs.^33^ ER stress‐mediated inhibition of TGF‐β signaling pathway inhibits EMT and migration of retinal pigment epithelial cells.^34^ Furthermore, ER stress inhibits EMT via calpain‐mediated C/EBP‐β and NF‐κB cleavage in gastric cancer.^35^ In addition, Qin et al found that ER stress suppresses cancer metastasis through inhibition of the AKT‐STAT3‐MMPs axis.^36^ Hence, ER stress is increasingly recognized as an anticancer mechanism and ER stress‐targeted therapy is expected to become a novel treatment strategy. In this study, we found that β‐as activates ER stress in BCa cells and confirmed the results by qRT‐PCR, Western blotting, and immunofluorescence experiments. Furthermore, we found that β‐as‐mediated inhibition of metastasis was associated with ER stress. Using an ER stress inhibitor, TUDCA, we confirmed that inhibition of ER stress attenuates β‐as‐induced metastatic inhibition in BCa.

As an active transcription factor located in the ER, ATF6 is involved in the development, progression, and metastasis of cancer.^37^ DP44MT, an anticancer agent, exerts anticancer effects by upregulating the expression of ATF6 in multiple tumor tissues.^38^ Activated‐ATF6 increases the expression of CHOP to transcriptionally upregulate ATG3 and ATG5 to clear EMT‐inducing transcription factors SNAI/snail and TWIST.^39^ A recent study reported that in chronic lymphoblastic leukemia, ATF6 is indispensable for DAPK1 expression, which activates autophagy to inhibit tumor metastasis.^40^ Spaan et al. demonstrated that up‐regulation of ATF6 not only activates the Perk‐EIF2α pathway, but also increases sensitivity to UPR activation, which suppresses cancer cell proliferation and stemness.^19^

When cleaved ATF6 enters the nucleus, it binds to the ER stress response elements in ER stress genes and up‐regulates downstream targets to enhance the function of ER.^41^ In the present study, we performed cytoplasmic protein separation assay to observe the intracellular distribution of ATF6, and found that β‐as activates ATF6 and induces its translocation into the nucleus, which was corroborated using immunofluorescence. To verify whether β‐as inhibits metastasis of BCa by activating the ATF6 branch of ER stress, we used siATF6 to knockdown ATF6 expression and found that it attenuated β‐as‐mediated inhibition of metastasis and EMT in BCa. This suggests that β‐as may be a potential targeted‐ATF6 drug. Taken together, our data suggest that β‐as suppresses the migration, invasion, and EMT of BCa by activating the ATF6 branch of ER stress. However, how ATF6 activates ER stress, and whether ATF6 affects the metastasis of BCa through other mechanisms following β‐as treatment remains to be explored further.

In conclusion, this study demonstrated that β‐as suppresses the invasion and migration of BCa by activating the ATF6 branch of ER stress. Our study not only demonstrates the anticancer effect of β‐as in BCa for the first time, but also provides a supporting underlying mechanism for the same. These findings provide new insights into the potential treatments for BCa.

## AUTHOR CONTRIBUTIONS

**Bo Liu:** Formal analysis (lead); writing – original draft (lead). **Weichao Dan:** Formal analysis (equal); methodology (lead). **Yi Wei:** Formal analysis (lead). **Yan Zhang:** Software (lead). **Chi Wang:** Software (equal). **Yuzeshi Lei:** Methodology (equal). **Tao Hou:** Methodology (equal). **Yong Zhang:** Writing – review and editing (equal). **Ye Gao:** Writing – review and editing (equal).

## FUNDING INFORMATION

No funding was received.

## CONFLICT OF INTEREST STATEMENT

The authors declare that they have no known competing financial interests or personal relationships that could have appeared to influence the work reported in this paper.

## ETHICS APPROVAL AND CONSENT TO PARTICIPATE

All animal care and experiments were authorized by the Institutional Animal Care and Use Committee of Xi'an Jiaotong University.

## PATIENT CONSENT FOR PUBLICATION

No patients participated in the study.

## Data Availability

All data generated or analyzed during this study are included in this published article.

## References

[1] Mahdavifar N , Ghoncheh M , Pakzad R , Momenimovahed Z , Salehiniya H . Epidemiology, incidence and mortality of bladder cancer and their relationship with the development index in the world. Asian Pac J Cancer Prev. 2016;17:381‐386. 26838243 10.7314/apjcp.2016.17.1.381

[2] Liu X , Jiang J , Yu C , et al. Secular trends in incidence and mortality of bladder cancer in China, 1990‐2017: a joinpoint and age‐period‐cohort analysis. Cancer Epidemiol. 2019;61:95‐103. 31176961 10.1016/j.canep.2019.05.011

[3] Babjuk M , Burger M , Compérat EM , et al. European association of urology guidelines on non‐muscle‐invasive bladder cancer (TaT1 and carcinoma in situ)–2019 update. Eur Urol. 2019;76(5):639‐657. 31443960 10.1016/j.eururo.2019.08.016

[4] Feifer AH , Taylor JM , Tarin TV , Herr HW . Maximizing cure for muscle‐invasive bladder cancer: integration of surgery and chemotherapy. Eur Urol. 2011;59:978‐984. 21257257 10.1016/j.eururo.2011.01.014 PMC3137649

[5] Kamat AM , Colombel M , Sundi D , et al. BCG‐unresponsive non‐muscle‐invasive bladder cancer: recommendations from the IBCG. Nat Rev Urol. 2017;14:244‐255. 28248951 10.1038/nrurol.2017.16

[6] Fang YQ , Shi C , Liu L , Fang RM . Analysis of transformation and excretion of β‐asarone in rabbits with GC‐MS. Eur J Drug Metab Pharmacokinet. 2012;37:187‐190. 22351074 10.1007/s13318-012-0083-z

[7] Wang N , Wang H , Li L , Li Y , Zhang R . β‐Asarone inhibits amyloid‐β by promoting autophagy in a cell model of Alzheimer's disease. Front Pharmacol. 2019;10:1529. 32009952 10.3389/fphar.2019.01529 PMC6979317

[8] Wang T‐L , Ouyang C‐S , Lin L‐Z . β‐Asarone suppresses Wnt/β‐catenin signaling to reduce viability, inhibit migration/invasion/adhesion and induce mitochondria‐related apoptosis in lung cancer cells. Biomed Pharmacother. 2018;106:821‐830. 29990876 10.1016/j.biopha.2018.07.009

[9] Tao H , Ding X , Wu J , et al. Asarone increases chemosensitivity by inhibiting tumor glycolysis in gastric cancer. Evid Based Complement Alternat Med. 2020;2020:6981520. 32351601 10.1155/2020/6981520 PMC7171649

[10] Liu L , Wang J , Shi L , et al. β‐Asarone induces senescence in colorectal cancer cells by inducing Lamin B1 expression. Phytomedicine. 2013;20:512‐520. 23357361 10.1016/j.phymed.2012.12.008

[11] Qi H , Chen L , Ning L , et al. Proteomic analysis of β‐asarone induced cytotoxicity in human glioblastoma U251 cells. J Pharm Biomed Anal. 2015;115:292‐299. 26263057 10.1016/j.jpba.2015.07.036

[12] Fei L , Huimei H , Dongmin C . Pivalopril improves anti‐cancer efficiency of cDDP in breast cancer through inhibiting proliferation, angiogenesis and metastasis. Biochem Biophys Res Commun. 2020;533:853‐860. 33008601 10.1016/j.bbrc.2020.07.059

[13] He Q , Shi J . MSN anti‐cancer nanomedicines: chemotherapy enhancement, overcoming of drug resistance, and metastasis inhibition. Adv Mater. 2014;26:391‐411. 24142549 10.1002/adma.201303123

[14] Cubillos‐Ruiz JR , Mohamed E , Rodriguez PC . Unfolding anti‐tumor immunity: ER stress responses sculpt tolerogenic myeloid cells in cancer. J Immunother Cancer. 2017;5:5. 28105371 10.1186/s40425-016-0203-4 PMC5240216

[15] Hotamisligil GS , Davis RJ . Cell signaling and stress responses. Cold Spring Harb Perspect Biol. 2016;8:a006072. 27698029 10.1101/cshperspect.a006072 PMC5046695

[16] Rashid H‐O , Yadav RK , Kim H‐R , Chae H‐J . ER stress: autophagy induction, inhibition and selection. Autophagy. 2015;11:1956‐1977. 26389781 10.1080/15548627.2015.1091141 PMC4824587

[17] Hillary RF , FitzGerald U . A lifetime of stress: ATF6 in development and homeostasis. J Biomed Sci. 2018;25:48. 29801500 10.1186/s12929-018-0453-1 PMC5968583

[18] Sharma RB , Darko C , Alonso LC . Intersection of the ATF6 and XBP1 ER stress pathways in mouse islet cells. J Biol Chem. 2020;295:14164‐14177. 32788214 10.1074/jbc.RA120.014173 PMC7549035

[19] Spaan CN , Smit WL , van Lidth JF , et al. Expression of UPR effector proteins ATF6 and XBP1 reduce colorectal cancer cell proliferation and stemness by activating PERK signaling. Cell Death Dis. 2019;10:490. 31227689 10.1038/s41419-019-1729-4 PMC6588629

[20] Liu F , Chang L , Hu J . Activating transcription factor 6 regulated cell growth, migration and inhibiteds cell apoptosis and autophagy via MAPK pathway in cervical cancer. J Reprod Immunol. 2020;139:103120. 32234634 10.1016/j.jri.2020.103120

[21] Sicari D , Fantuz M , Bellazzo A , et al. Mutant p53 improves cancer cells' resistance to endoplasmic reticulum stress by sustaining activation of the UPR regulator ATF6. Oncogene. 2019;38:6184‐6195. 31312025 10.1038/s41388-019-0878-3

[22] Lamouille S , Xu J , Derynck R . Molecular mechanisms of epithelial‐mesenchymal transition. Nat Rev Mol Cell Biol. 2014;15:178‐196. 24556840 10.1038/nrm3758 PMC4240281

[23] Limia CM , Sauzay C , Urra H , Hetz C , Chevet E , Avril T . Emerging roles of the endoplasmic reticulum associated unfolded protein response in cancer cell migration and invasion. Cancers (Basel). 2019;11:631. 31064137 10.3390/cancers11050631 PMC6562633

[24] Zheng Y , Liu P , Wang N , et al. Betulinic acid suppresses breast cancer metastasis by targeting GRP78‐mediated glycolysis and ER stress apoptotic pathway. Oxid Med Cell Longev. 2019;2019:8781690. 31531187 10.1155/2019/8781690 PMC6721262

[25] Zhang B , Han H , Fu S , et al. Dehydroeffusol inhibits gastric cancer cell growth and tumorigenicity by selectively inducing tumor‐suppressive endoplasmic reticulum stress and a moderate apoptosis. Biochem Pharmacol. 2016;104:8‐18. 26774454 10.1016/j.bcp.2016.01.002

[26] Shenvi S , Diwakar L , Reddy GC . Nitro derivatives of naturally occurringβ‐Asarone and their anticancer activity. Int J Med Chem. 2014;2014:835485. 25383224 10.1155/2014/835485 PMC4207380

[27] Pastushenko I , Blanpain C . EMT transition states during tumor progression and metastasis. Trends Cell Biol. 2019;29:212‐226. 30594349 10.1016/j.tcb.2018.12.001

[28] Li L , Wu M , Wang C , et al. β‐Asarone inhibits invasion and EMT in human glioma U251 cells by suppressing splicing factor HnRNP A2/B1. Molecules. 2018;23:671. 29547514 10.3390/molecules23030671 PMC6017590

[29] Dicks N , Gutierrez K , Michalak M , Bordignon V , Agellon LB . Endoplasmic reticulum stress, genome damage, and cancer. Front Oncol. 2015;5:11. 25692096 10.3389/fonc.2015.00011 PMC4315039

[30] Wu H , Li J , Guo E , Luo S , Wang G . MiR‐410 acts as a tumor suppressor in estrogen receptor‐positive breast cancer cells by directly targeting ERLIN2 via the ERS pathway. Cell Physiol Biochem. 2018;48:461‐474. 30016800 10.1159/000491777

[31] Kania E , Pająk B , Orzechowski A . Calcium homeostasis and ER stress in control of autophagy in cancer cells. Biomed Res Int. 2015;2015:352794. 25821797 10.1155/2015/352794 PMC4363509

[32] Chen Z , Zhang J , Xue H , et al. Nitidine chloride is a potential alternative therapy for glioma through inducing endoplasmic reticulum stress and alleviating epithelial‐mesenchymal transition. Integr Cancer Ther. 2020;19:1534735419900927. 32129091 10.1177/1534735419900927 PMC7057402

[33] Bandyopadhyay S , Pai SK , Gross SC , et al. The Drg‐1 gene suppresses tumor metastasis in prostate cancer. Cancer Res. 2003;63:1731‐1736. 12702552

[34] Ouyang S , Ji D , He S , Xia X . Endoplasmic reticulum stress as a novel target to inhibit transdifferentiation of human retinal pigment epithelial cells. Front Biosci (Landmark ed). 2022;27:38. 35226981 10.31083/j.fbl2702038

[35] Wu S‐M , Lin W‐Y , Shen C‐C , et al. Melatonin set out to ER stress signaling thwarts epithelial mesenchymal transition and peritoneal dissemination via calpain‐mediated C/EBPβ and NFκB cleavage. J Pineal Res. 2016;60:142‐154. 26514342 10.1111/jpi.12295

[36] Wu J , Xiao Z , Li H , et al. Present status, challenges, and prospects of dihydromyricetin in the Battle against cancer. Cancers (Basel). 2022;14:3487. 35884547 10.3390/cancers14143487 PMC9317349

[37] Coleman OI , Lobner EM , Bierwirth S , et al. Activated ATF6 induces intestinal dysbiosis and innate immune response to promote colorectal tumorigenesis. Gastroenterology. 2018;155:1539‐1552.e12. 30063920 10.1053/j.gastro.2018.07.028

[38] Merlot AM , Shafie NH , Yu Y , et al. Mechanism of the induction of endoplasmic reticulum stress by the anti‐cancer agent, di‐2‐pyridylketone 4,4‐dimethyl‐3‐thiosemicarbazone (Dp44mT): activation of PERK/eIF2α, IRE1α, ATF6 and calmodulin kinase. Biochem Pharmacol. 2016;109:27‐47. 27059255 10.1016/j.bcp.2016.04.001

[39] Kaarniranta K , Blasiak J , Liton P , Boulton M , Klionsky DJ , Sinha D . Autophagy in age‐related macular degeneration. Autophagy. 2023;19:388‐400. 35468037 10.1080/15548627.2022.2069437 PMC9851256

[40] Gade P , Kimball AS , DiNardo AC , et al. Death‐associated protein Kinase‐1 expression and autophagy in chronic lymphocytic leukemia are dependent on activating transcription Factor‐6 and CCAAT/enhancer‐binding protein‐β. J Biol Chem. 2016;291:22030‐22042. 27590344 10.1074/jbc.M116.725796 PMC5063986

[41] Hong M , Luo S , Baumeister P , et al. Underglycosylation of ATF6 as a novel sensing mechanism for activation of the unfolded protein response. J Biol Chem. 2004;279:11354‐11363. 14699159 10.1074/jbc.M309804200

